# Improved Electrical Properties of Organic Modified Thermoplastic Insulation Material for Direct Current Cable Application

**DOI:** 10.3390/polym16010046

**Published:** 2023-12-22

**Authors:** Yunpeng Zhan, Xu Yang, Jiaming Yang, Shuai Hou, Mingli Fu

**Affiliations:** 1 Electric Power Research Institute, China Southern Power Grid, Guangzhou 510663, China; houshuai@csg.cn (S.H.); fuml@csg.cn (M.F.); 2 Key Laboratory of Engineering Dielectrics and Its Application, Harbin University of Science and Technology, Harbin 150080, China; jmyang@hrbust.edu.cn

**Keywords:** polypropylene, organic modification, DC properties, density functional theory calculation, bipolar charge transport model

## Abstract

To achieve exceptional recyclable DC cable insulation material using thermoplastic polypropylene (PP), we have introduced the organic polar molecule styrene-maleic anhydride copolymer (SMA) into PP-based insulation materials following the principles of deep trap modification. PP, PP/SMA, PP/ethylene-octene copolymer (POE), and PP/POE/SMA insulating samples were prepared, and their meso-morphology, crystalline morphology, and molecular structure were comprehensively characterized. The results indicate that SMA can be uniformly dispersed in PP with minimal impact on the crystalline morphology of PP. The DC electrical properties of the materials were tested at temperatures of 30, 50, and 70 °C. The findings demonstrate that the introduction of SMA can improve the DC properties of the material in both PP and PP/POE. The thermal stimulated depolarization current results reveal that SMA can introduce deep traps into the material, thereby improving its DC properties, which is in agreement with the quantum chemical calculation results. Subsequently, a bipolar carrier transport model was employed for coaxial cables to simulate the space charge distribution in the insulation layer of the four sets of insulation samples as well as the actual cable in service. The results highlight that SMA can significantly suppress space charge in PP and PP/POE systems, and it exhibits excellent electric field distortion resistance. In summary, the results illustrate that SMA is expected to be used as an organic deep trap modifier in PP-based cable insulation materials.

## 1. Introduction

In recent years, with the widespread application of clean energy sources like solar and wind power, there has been an increasing demand for long-distance power transmission systems capable of delivering electricity from remote locations, such as mountains or offshore sites, to urban areas [1,2]. High-voltage direct current (HVDC) systems are the preferred choice for long-distance high-power transmission due to their cost-effective transmission lines, rapid regulation capabilities, operational stability, and unlimited transmission distance. With the integration of fully controlled power electronic devices, flexible HVDC transmission has addressed the reactive power compensation issues of traditional HVDC systems, laying a foundation of reducing power supply cost and increasing transmission power. As one of the key components in flexible transmission systems, HVDC cables with excellent insulation performances are crucial to ensuring the stability and security of the transmission system [3,4,5,6].

Cross-linked polyethylene (XLPE) has conventionally served as a primary insulating material in the production of HVDC cables. However, it necessitates a high-temperature cross-linking process (generally peroxide-induced) to meet the demand of thermal stability. Additionally, the disposal of retired XLPE cables poses environmental challenges [7]. Therefore, it is imperative to develop environmentally friendly recyclable insulation materials for HVDC cables. Polypropylene (PP), known for its high melting point, exceptional electrical properties, and recyclability, emerges as the top choice to replace XLPE in environmentally conscious HVDC cable insulation materials [8,9,10,11,12].

The primary challenge in developing insulation materials for HVDC cables lies in addressing the accumulation of space charges [13,14,15]. A substantial buildup of space charge can severely distort the electric field within the insulation layer, serving as a significant hindrance to the development of HVDC cables. Improvements in the dielectric properties of PP insulating materials mainly revolve around nano-particle composite modification technology and grafting modification technology. Nanoparticle composite’s effectiveness relies on its ability to achieve a uniform dispersion of nanoparticle [14,16]. However, due to the poor dispersion of nanoparticles in non-polar solid polymers, the aggregated nanoparticles can become impurities and degrade insulation quality. Research by N. Quirke et al. utilizing density functional theory (DFT), demonstrates that both physical and chemical defects can generate deep traps in polymers, thereby improving their DC properties [17]. Consequently, based on the concept of introducing deep traps to counteract space charge accumulation in nanocomposites, Zhou et al. grafted maleic anhydride (MAH) onto PP to enhance its DC properties [18]. Nonetheless, whether through heat-initiated or photo-initiated grafting, chemical grafting inevitably leads to some degree of irreversible damage to PP. Moreover, the grafting reaction process is complex, with factors such as reaction temperature, monomer content, and reaction time affecting the effectiveness of modification [19,20,21].

In this study, a styrene-maleic anhydride copolymer (SMA) was used as a deep trap modification additive to disperse in a polymer through melt blending. PP, PP/POE, PP/SMA, and PP/POE/SMA were prepared by modifying the insulation materials of PP and PP composite elastomer ethylene-octene copolymer (POE), respectively. The effects of SMA on the DC electrical properties were studied. Furthermore, the deep trap introduction function of SMA was investigated using quantum chemical calculation and the thermal depolarization current. The impact of SMA modification on the space charge distribution in the HVDC cable was further validated by employing bipolar charge transport modelling.

## 2. Materials and Methods

### 2.1. Materials and Preparation

**Figure 1:**
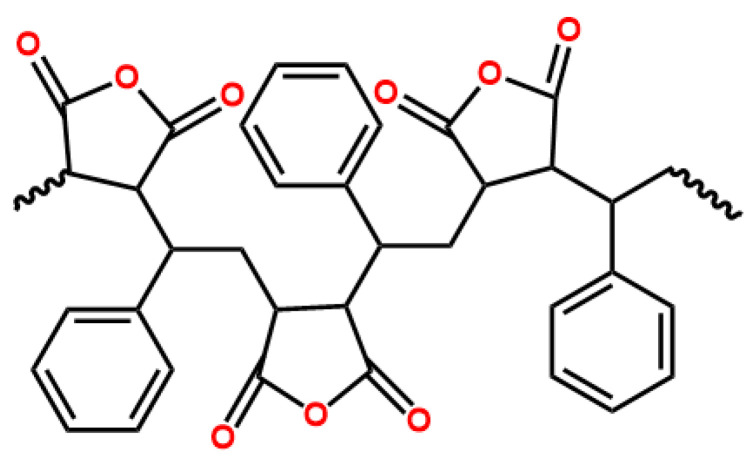
Molecular structure of SMA.

PP produced by Borealis(Abu Dhabi, United Arab Emirates) (SC876F; melting point, 149 °C; density, 0.9 g/cm^3^; melt flow rate, 3.8 g/10 min) was selected as the base polymer. The elastomer POE (LC170), produced by LG Company, has a melting point of 58 °C, density of 0.87 g/cm^3^, and melt flow rate of 1.1 g/10 min. The SMA was manufactured by Sigma-aldrich (Shanghai, China), with a melting point of 160 °C and a density of 1.1 g/cm^3^. Its molecular structure is shown in Figure 1.

The insulating composite samples were prepared using the following method: PP and POE pellets were weighed according to certain proportions, as shown in Table 1. The dosage for POE and SMA can be found in references [9,21]. The material was blended using a torque rheometer, and the mixing time lasted 5 min under a temperature of 190 °C with a rotation speed of 60 r/min. According to specific requirements of the different experiments, different sizes of mold were selected. By hot pressing at 190 °C, the samples were prepared into different shapes which met the requirements of the subsequent experiments. The samples were then placed in a plate curing press with a pressure of 5 MPa which increased every 5 min until the pressure reached 15 MPa and then cooled for 5 min at a pressure of 15 MPa. At the end, the samples were removed from the mold. Finally, the samples were placed in a vacuum oven at 80 °C for 48 h to eliminate residual stress moisture.

### 2.2. Characterization

A scanning electron microscope (SEM) produced by Hitachi (Tokyo, Japan) was used to observe the mesoscopic morphology of the materials. The materials were etched by saturated long-chain alkane n-heptane, and the elastomer phase was removed. Only the higher molecular weight PP phase and SMA were retained. Samples with a thickness of 1 mm were treated with low-temperature (−180 °C) brittle fracture, and the sections were etched with N-heptane for 15 min at 60 °C. Then, the sections were ultrasonically cleaned with deionized water. After they were dried, the sections were sprayed with gold, and the structures were observed using SEM. The scanning conditions of SEM included an acceleration electric field of 5 kV, a probe current of 5 mA, and a probe height of 8 mm.

In order to further study the effects of SMA on the crystallization morphology of PP and PP/POE, the non-isothermal crystallization processes of the composites were observed using XPF500C polarized light microscopy (POM) produced by Carl Zeiss in Shanghai, China. A small sample was placed on a hot plate and heated to 220 °C at a rate of 0.5 °C/s for 5 min, then cooled to 90 °C at a rate of 0.1 °C/s. During the cooling process, the crystallization process of the sample was recorded.

The molecular structure of the modified material was characterized using Fourier transform infrared spectroscopy (FT-IR). The FT/IR6100 spectrometer was produced by JIASKE (Shanghai, China) Trading Co., Ltd. The transmission mode was used in the test. The range of wave numbers was from 4000 cm^−1^ to 600 cm^−1^, the resolution was 2.0 cm^−1^, the number of scanning times was 32, and the thickness of the samples was 100 μm.

### 2.3. Direct Current Electrical Properties

The distribution of space charge in different temperature fields was measured using electro-acoustic pulse (PEA) PEA-01 produce by Heyi (Shanghai, China). The upper electrode and the lower electrode were both aluminum electrodes, and a semi-conductive layer was added between the upper electrode and the sample to ensure the matching of acoustic impedance. The pulse power supply amplitude was 500 V, with a width of 10 ns. The samples were polarized for 1800 s and depolarized for 1800 s under a 40 kV/mm DC electric field. The test temperatures were 30 °C, 50 °C, and 70 °C.

According to the electric field strength and temperature of the insulation layer of the high-voltage DC cable, the conductance currents of the four groups of materials at 30 °C, 50 °C, and 70 °C with an electric field strength of 10 kV/mm–45 kV/mm were measured. The diameter of the high-voltage electrode was 76 mm, the outer diameter of the protective electrode was 74 mm, the inner diameter of the protective electrode was 54 mm, and the diameter of the measuring electrode was 50 mm. The test adopted the step-by-step voltage boosting method. The sample and electrode were placed in the shielding box in a constant temperature box. The conductance current was measured using an Est122 picoammeter produce by Penglichi (Beijing, China).

In the DC breakdown test, the diameter of the high-voltage electrode was 35 mm and the diameter of the grounding electrode was 50 mm. The electrode and the sample were placed into a glass container and filled with insulating oil during the test to avoid surface flashover. Before the test, the oil and the electrode were preheated using a heater attached to the outer bottom of the vessel. The temperature was set to 30 °C, 50 °C, and 70 °C, and the voltage was increased at a rate of 500 V/s. More than 10 experiments were carried out for each sample, and the DC breakdown strength was characterized by its two-parameter Weibull distribution.

The trap level distribution characteristics were determined by the thermally stimulated depolarization current (TSDC). The samples with a thickness of 100 μm were polarized at 80 °C for 30 min under 40 kV/mm. Then, the samples were quickly cooled to −20 °C. Next, the polarizing electric field was removed, and the samples were short-circuited for 5 min. Finally, the samples were heated to 160 °C at a heating rate of 3 °C/min, and the depolarization current was recorded using an electrometer.

### 2.4. Density Functional Theory Calculation

The DFT method was used to calculate the quantum chemical properties of the materials. Based on the B3LYP hybrid density functional method, a PP molecule with a polymerization degree of 5 and one SMA molecule with a structure unit were constructed, and their structures were optimized at a 6–31 G(d) set, which consists of six atomic orbitals (s, p, d orbitals) that are represented by a combination of Gaussian-type functions. The influence of its quantum chemical properties on its electrical properties was analyzed. Because both POE and PP are saturated alkane polymers containing only C and H atoms with a carbon skeleton and their electronic structures are similar, they are not calculated separately.

### 2.5. Carrier Transport Simulation

The space charge distribution based on the bipolar charge transport model was stimulated in the cable insulation. The transport of the charge materials was described by the current continuity equation, Poisson equation, and conduction equation. The injection of electrons and holes from the electrodes was described by the Schottky equation. The charge source term can be used to describe the processes of trapping, detrapping, and recombination. The temperature field was constructed by the solid heat transfer equation. Due to the limited space of this paper, the reason of the parameters selection is detailed in the references [22,23]. The parameters of the carrier transport simulation are shown in Table 2.

## 3. Results

### 3.1. Characterization

#### 3.1.1. Microscopic Morphology

**Figure 2:**
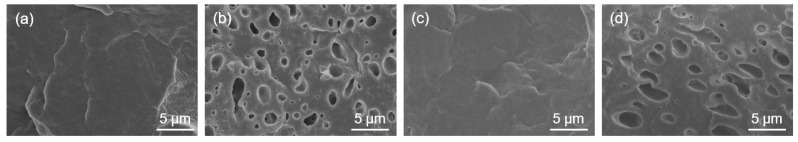
SEM results for PP and modified PP. (**a**) SEM result for PP, (**b**) SEM result for PP/POE, (**c**) SEM result for PP/SMA, and (**d**) SEM result for PP/POE/SMA.

The SEM results are shown in Figure 2. The holes shown in Figure 2 are formed after the elastomer is etched. Compared with pure PP, as shown in Figure 2b, the PP/POE material has an obvious island structure, the POE phase is ellipsoidal and distributed in the PP, and the particle size varies from 0.5 μm to 3 μm. By comparing the SEM of the materials before and after adding SMA, as shown in Figure 2c,d, it can be seen that there is no significant difference between the meso-morphology of the materials before and after adding SMA. Different from inorganic nano-particles, all-organic modified filler SMA exhibits better compatibility with polymer macromolecules and can be uniformly dispersed in the material without agglomeration behavior [24]. Comparing PP/POE with the PP/POE/SMA shown in Figure 2b,d, one can see that the size of the POE phase in the PP/POE/SMA has slightly increased, but its distribution is more uniform. This indicates that the introduction of SMA has little effect on the mesoscopic morphology of the material.

#### 3.1.2. Crystal Morphology

**Figure 3:**
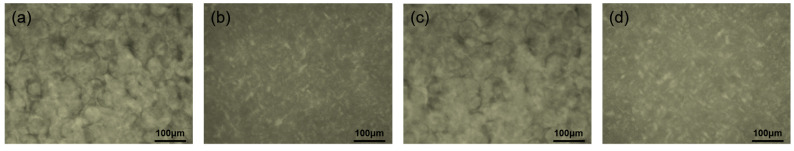
POM results for PP and modified PP. (**a**) POM result for PP, (**b**) POM result for PP/POE, (**c**) POM result for PP/SMA, and (**d**) POM result for PP/POE/SMA.

The POM results are shown in Figure 3. As shown in Figure 3a, a distinct black cross extinction can be observed in PP, indicating the presence of a spherulite structure with optical anisotropy and a regular spherulite with a well-defined profile. The spherulite size of neat PP is between 50–70 μm. As shown in Figure 3c, the crystallization morphology of PP/SMA is not significantly different from that of PP, indicating that SMA does not affect the crystallization behavior of PP. Figure 3b,d shows the crystal morphology of PP/POE and PP/POE/SMA, respectively. After adding the elastomer POE, the spherulite size of the blends become smaller, the black cross extinction profile becomes blurred, and the spherulite becomes finer without obvious an boundary.

#### 3.1.3. Molecular Structure

**Figure 4:**
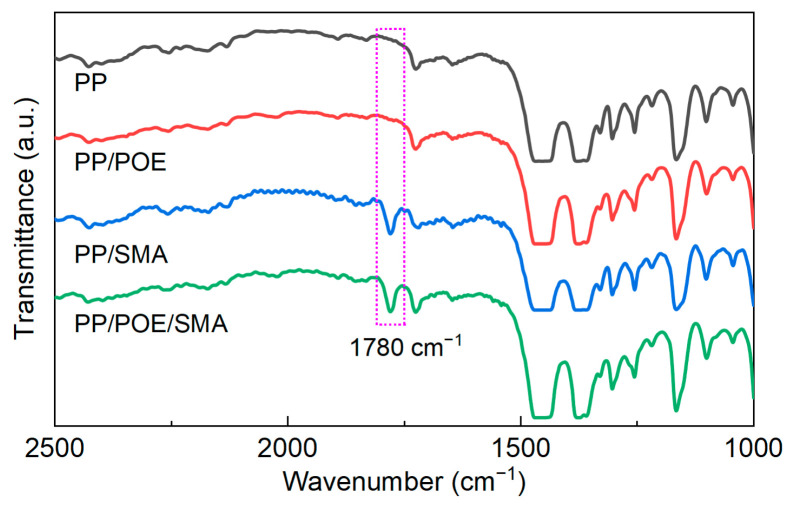
FT-IR results for PP and modified PP.

After vacuum treatment at 80 °C for 48 h, the FT-IR results of each material are shown in Figure 4. After doping with SMA, new characteristic absorption peaks of the PP/SMA and PP/POE/SMA appeared at 1780 cm^−1^, corresponding to the stretching vibration absorption peak of the carbonyl group on the MAH segment of the SMA [19]. It is verified that SMA can be blended in PP and PP/POE, and it still exists stably after high-temperature vacuum treatment.

### 3.2. Direct Current Electrical Properties

#### 3.2.1. Space Charge Distribution

**Figure 5:**
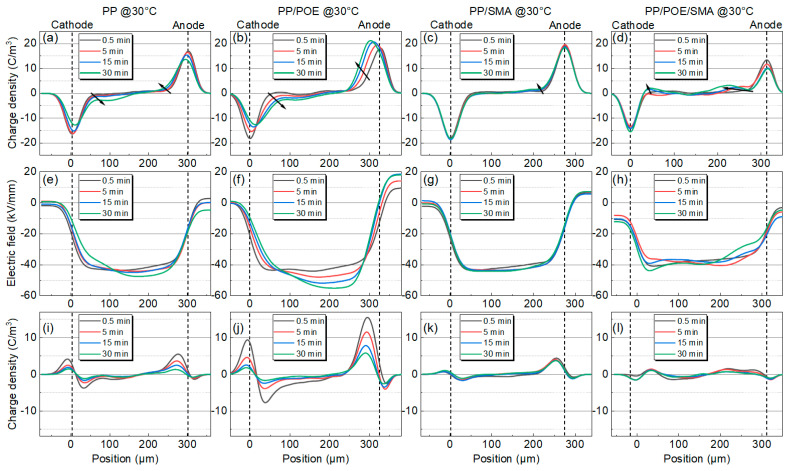
Space charge test results for PP and modified PP at 30 °C. (**a**–**d**) Space charge distribution, (**e**–**h**) electric field distribution during the polarization process, (**i**–**l**) space charge distribution during the short-circuit process. (**a**,**e**,**i**) PP. (**b**,**f**,**j**) PP/POE. (**c**,**g**,**k**) PP/SMA. (**d**,**h**,**l**) iPP/POE/SMA.

**Figure 6:**
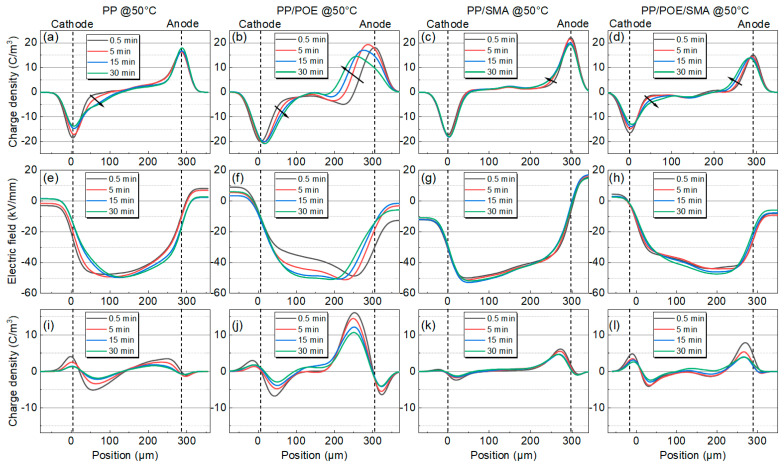
Space charge test results for PP and modified PP at 50 °C. (**a**–**d**) Space charge distribution, (**e**–**h**) electric field distribution during the polarization process, (**i**–**l**) space charge distribution during the short-circuit process. (**a**,**e**,**i**) PP. (**b**,**f**,**j**) PP/PE. (**c**,**g**,**k**) PP/SMA. (**d**,**h**,**l**) PP/POE/SMA.

**Figure 7:**
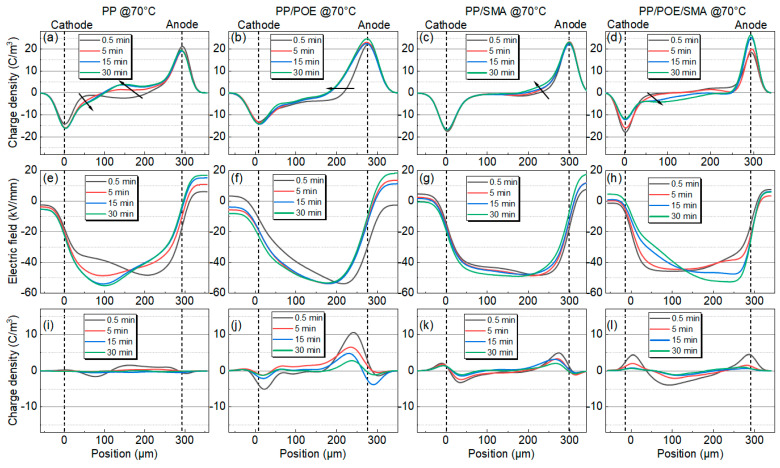
Space charge test results for PP and modified PP at 70 °C. (**a**–**d**) Space charge distribution, (**e**–**h**) electric field distribution during the polarization process, (**i**–**l**) space charge distribution during the short-circuit process. (**a**,**e**,**i**) PP. (**b**,**f**,**j**) PP/PE. (**c**,**g**,**k**) PP/SMA. (**d**,**h**,**l**) PP/POE/SMA.

The results of the space charge measurements at 30, 50, and 70 °C are shown in Figure 5, Figure 6 and Figure 7; from the top to the bottom are the space charge distribution curve in the polarization stage, the electric field distribution curve in the polarization stage, and the space charge distribution curve in the short-circuit depolarization stage. The accumulated space charge in the sample mainly comes from two parts. One part is that the trapped carriers or transportable carriers produced via Schottky injection become the homocharge. The other part is the heterocharges caused by the ionization and migration of organic or inorganic impurities under the action of the electric field [25]. As shown in Figure 5a, in the neat PP at 30 °C, homocharge accumulation appears in the sample, and the charge injection increases with the increase in the polarization process. After adding the elastomer POE, as shown in Figure 5b, the amount of charge injected by the electrode increases significantly, and the phenomenon of charge packet migration occurs. The electric field in the sample was seriously distorted to 57 kV/mm. As shown in Figure 5c, the charge injection in the PP/SMA is significantly improved with the addition of SMA, and the electric field distortion is significantly less than that of the unmodified PP. Figure 5d shows the space charge distribution characteristics of PP/POE/SMA. Compared with Figure 5b, the charge injection phenomenon is significantly improved after modification, and the stray charge and electric field distortion are also significantly restrained. The maximum electric field is only 45 kV/mm.

When the test temperature rises to 50 °C, the charge injection phenomenon in all the samples increases to varying degrees. With the polarization process, the charge packets migrate to the middle of the samples, and the depth of charge injection increases gradually. As shown in Figure 6a, the amount of charge injected into the neat PP increases significantly, and the injection depth increases compared with that at 30 °C. After blending with POE, there is more space charge accumulated in the PP/POE, as shown in Figure 6b. As shown in Figure 6c,d, although the charge injection is slightly increased compared with 30 °C, compared with unmodified PP and PP/POE, the charge injection in the SMA-modified samples is significantly improved, and the depth of charge injection is significantly reduced.

With an increase in the test temperature, the charge injection is further aggravated at 70 °C, as shown in Figure 7a. A large amount of charge is injected at the initial stage of polarization, and the positive charge density injected by the anode is much higher than the negative charge density injected by the cathode. The electric field inside the sample is seriously distorted, and the maximum field strength reaches 56 kV/mm. As shown in Figure 7b, the charge injection rate in the PP/POE is significantly increased, reaching the inner part of the sample in 15 min, and the positive and negative charge packets meet the inner part of the sample. As shown in Figure 7c, only positive charge injection can be observed in the PP/SMA, and the amount of positive charge increases slightly. The distribution of the space charge in the PP/POE/SMA is also improved compared with that of the PP/POE before modification; however, a large amount of homopolymer charge is still accumulated within the sample.

It is not difficult to compare the space charge characteristics of the four groups of materials at different temperatures. It is found that adding POE will increase the space charge accumulation, and SMA can suppress the space charge accumulation. The addition of elastomer can disturb the original crystalline structure of PP and increase the spherulite boundary. On the other hand, the introduction of the POE also increases the free volumes in the material, and the charge is transferred more easily in these free volumes. The addition of SMA as a deep trap introducer can form new localized energy levels into the material to capture charges. These deep traps near the electrodes will form a charge lattice and generate a Cullen shielding field after the trapped charges. As a result, the charge injection in the PP/SMA and PP/POE/SMA is significantly inhibited, and the space charge in the PP/SMA and PP/POE/SMA is obviously improved.

#### 3.2.2. Conductance Current

**Figure 8:**
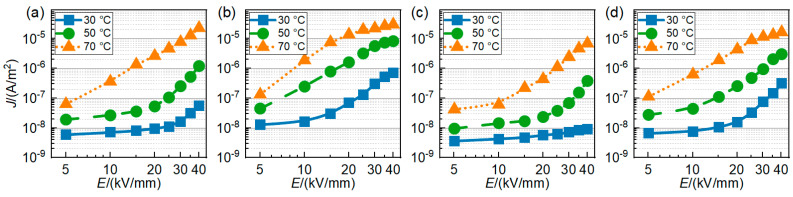
*E*–*J* curves for PP and modified PP at 30–70 °C. (**a**–**d**) PP, PP/POE, PP/SMA, and PP/POE/SMA, respectively.

The *E*–*J* curves of the four groups of materials at different temperatures are shown in Figure 8. As shown in Figure 8a, in the PP’s conductance characteristic curves, there are obvious threshold points shown at 30 and 50 ° C. According to the space charge limited current (SCLC) theory, this point is the threshold electric field at which the conductance mechanism changes from the Ohm region to the trap-limited SCLC (TLSCLC) region [26,27,28]. When the applied field is higher than the threshold strength, the space charge accumulation begins to appear in the sample. With increasing temperature, the threshold field moves towards a low electric field, and at 70 °C, the threshold field disappears in the test range. As shown in Figure 8b, the threshold field of PP/POE decreases significantly after the addition of the elastomer. At 50 °C, a transition from TLSCLC to trap-free SCLC (TFSCLC) can be observed, and the threshold field of the transition from TLSCLC to TFSCLC further decreases. As shown in Figure 8c, the introduction of SMA increases the threshold field of the charge injection while decreasing the material’s conductance current, which corresponds to the space charge test results in Figure 5, Figure 6 and Figure 7. Even at 70 °C, TFSCLC cannot be found within the range of the tested field; that is, the traps are not filled, indicating that SMA can introduce a large number of deep traps to improve the DC properties of the materials. In the PP/POE/SMA, as shown in Figure 8d, a transition from TLSCLC to TFSCLC occurs at 70 °C, illustrating the presence of a large amount of space charge in the material. All the traps have been filled, which is consistent with the space charge test results in Figure 8d. The addition of the elastomer increases the conductance current in the PP/POE significantly at all temperatures. The addition of SMA can evidently suppress the conductance current. Even at 70 °C, the conductance current of the SMA-modified material is still greatly reduced, and the threshold field moves toward the high electric field.

#### 3.2.3. Breakdown Strength

**Figure 9:**
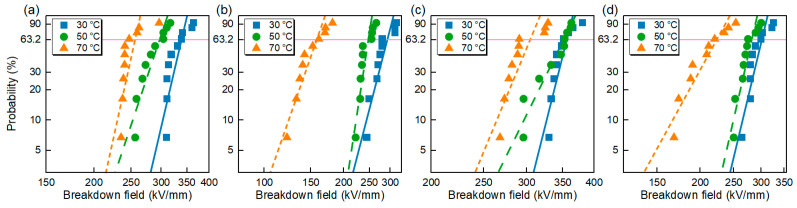
The Weibull distribution of DC breakdown strength for PP and modified PP at 30–70 °C. The characteristic breakdown strength is indicated by the purple line at a probability of 63.2%. (**a**) PP, (**b**) PP/POE, (**c**) PP/SMA, (**d**) PP/POE/SMA.

The results of the DC breakdown strength tests are shown in Figure 9. The data were calculated according to Weibull distribution statistical method, and the statistical results are shown in Table 3. *E*_b_ is the characteristic breakdown strength and *β* is the shape parameter. Based on the results, it can be seen that the breakdown field strength of each group of samples gradually decreases with increasing temperature. At 70 °C, the breakdown strength of the PP/POE and PP/POE/SMA is significantly decreased, which may be related to the melting point of the elastomer at 58 °C. The melting phase reduces the strength of the composite significantly. Compared with PP and PP/POE, the addition of SMA can improve the breakdown strength significantly, and the shape parameters of the modified materials are also improved, indicating that the reliability of the materials was improved.

#### 3.2.4. Thermal-Stimulated Depolarization Current

**Figure 10:**
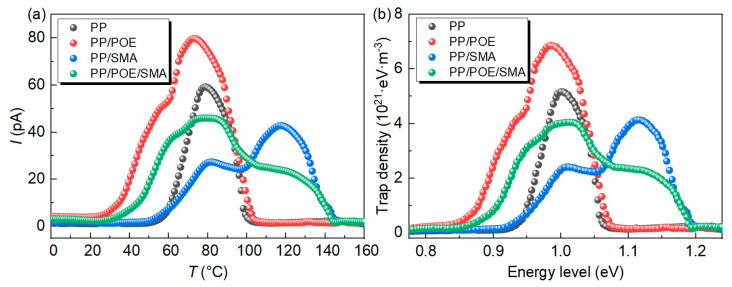
TSDC results for PP and modified PP. (**a**) Depolarization curves vs. temperature, (**b**) calculated trap energy distribution base on the depolarization curves.

Figure 10a shows the TSDC test results. According to the calculation method in reference [29], the trap energy level distribution is shown in Figure 10b. The charge release peaks of the four samples appear at 78 °C, which corresponds to a trap energy level of approximately 1.09 eV. This charge trap is formed by the intrinsic structure defect of the interface between the PP crystal region and the amorphous region [30]. After blending with POE, a new charge release peak appears near 50 °C, corresponding to the shallow trap introduced by POE, and the energy level is approximately 0.92 eV. After the introduction of SMA, the charge release peak caused by the intrinsic structure defect in the material decreases greatly, a new current release peak appears at 120 °C in the high-temperature region, and the trap depth corresponds to 1.12 eV. The new localized state level is introduced into the PP band by the polar group, which is consistent with the results of the space charge and conductance current measurements.

### 3.3. Density Functional Theory Calculation

**Figure 11:**
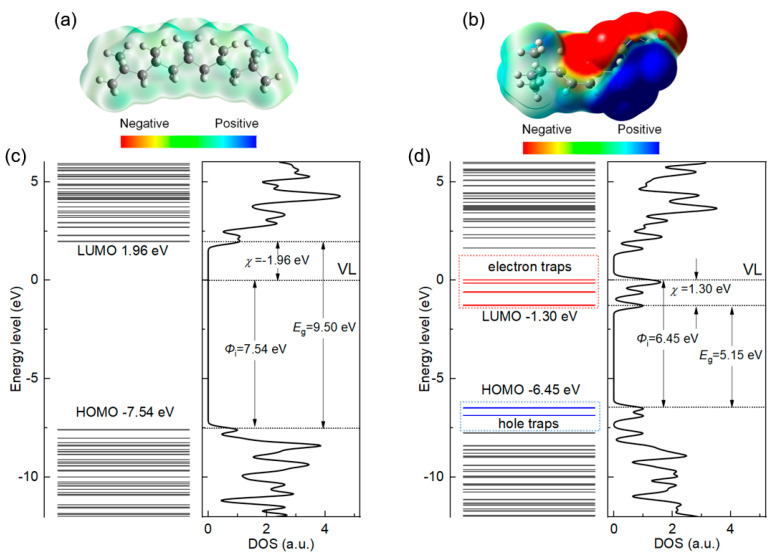
DFT calculation results for PP and modified PP. (**a**,**b**) Electrostatic potential for PP and SMA, respectively. (**c**,**d**) Electron structure for PP and SMA, respectively.

Figure 11a,b shows the electrostatic potential distribution of PP and SMA, respectively. The warm color represents negative electrostatic potentials, which can attract and trap electrons, while the cool color represents positive electrostatic potentials, which can attract and trap holes [30,31]. PP is a non-polar material that exhibits weak charge adsorption ability. As shown in Figure 11b, in the C=O structure, the O atom exhibits a strong negative electrostatic potential, which corresponds to the properties of the C=O electron acceptor. The five-membered ring structure of MAH and the continuous distribution of electronegative groups occupy one side of the five-membered ring, while the weakly adsorbed C atom is located on the other side of the five-membered ring. As a result, the C atom on the MAH is forced to become an electron donor under the action of the continuously distributed O atom on the opposite side, showing a positive electrostatic potential [19]. Figure 11c shows the calculated results of the electronic structure of PP. It can be seen that the band gap of PP is 9.5 eV and the electron affinity is negative, demonstrating the repulsive electron property. The ionization potential of PP is 7.54 eV, which is very difficult to ionize. As shown in Figure 11d, the band gap of SMA is significantly lower than that of PP, but it is still an insulator with an electron affinity of 1.3 eV. This indicates that SMA has a certain electron-absorbing ability in order to improve the DC performance of PP. The DFT calculation results are consistent with the TSDC test results shown in Figure 10.

### 3.4. Carrier Transport Simulation

**Figure 12:**
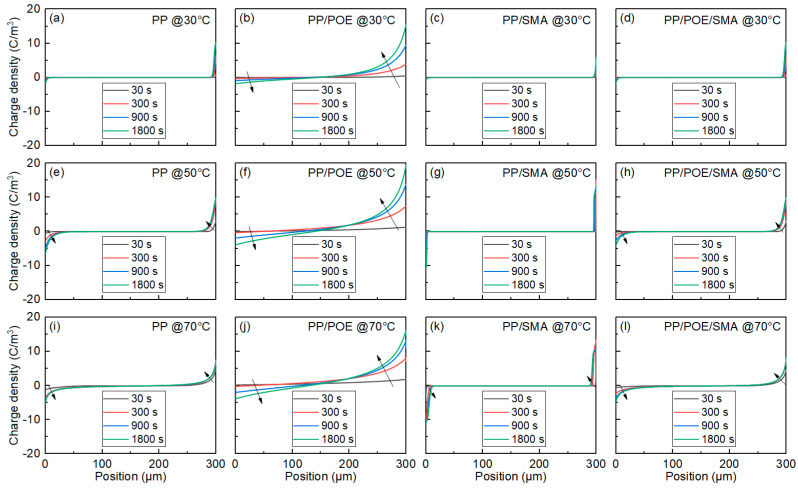
Simulation results of space charge distribution in the plate samples. (**a**–**d**) the space charge distribution of PP, PP/POE, PP/SMA, and PP/POE/SMA at 30 °C, respectively. (**e**–**h**) the space charge distribution of PP, PP/POE, PP/SMA, and PP/POE/SMA at 50 °C, respectively. (**i**–**l**) the space charge distribution of PP, PP/POE, PP/SMA, and PP/POE/SMA at 70 °C, respectively.

In order to simulate the structure of the coaxial cable, considering the effects of the temperature gradient on the charge injection and transfer, the space charge distribution and electric field distribution of the cable insulation are simulated using the bipolar carrier transport model. By comparing the simulation results with the experimental results, the parameters are optimized continuously, and the appropriate parameters are selected from the range of parameters. Referring to the TSDC test results, the definitions of four different sets of material trap parameters are given in Table 4. In PP/POE/SMA, according to the energy level, the shallowest level is defined as a shallow trap, and the deepest level is defined as a deep trap. Based on the parameters shown in Table 4, a bipolar carrier transport model was constructed to simulate the charge transport process and space charge distribution of four different materials as 300 μm plate insulating materials. The results are shown in Figure 12.

As shown in Figure 12, the charge injection increases with increasing temperature. Similar to the experimental results, as shown in Figure 12b, serious charge injection can be observed in the PP/POE, and the charge injection rate and depth increase with increasing temperature. When the temperature reaches 70 °C, the charge is injected into the materials within 300 s and reaches a steady state, which is consistent with the PEA test. After the introduction of SMA, the space charge in the material is significantly suppressed, and the depth of charge injection is significantly reduced. The simulation results are consistent with the experimental phenomena, and the reliability of the model and the selection of the parameters is verified.

**Figure 13:**
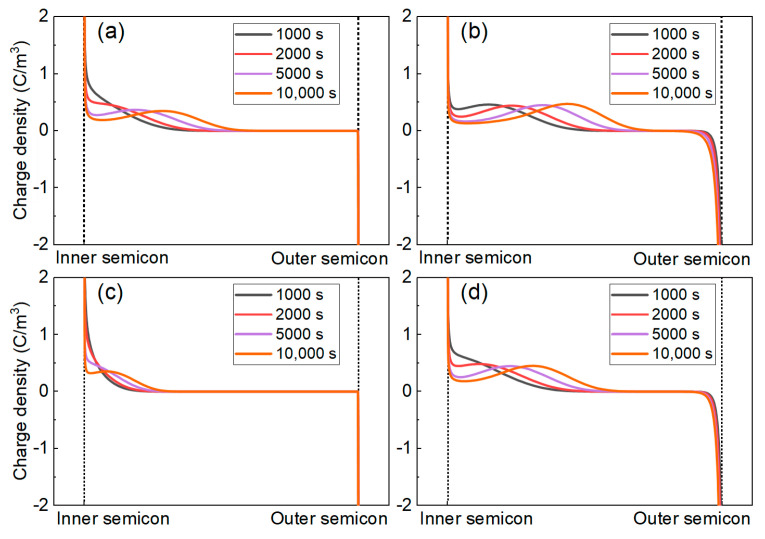
Simulation results of the space charge distribution in the cable insulation. (**a**–**d**) PP, PP/POE, PP/SMA, and PP/POE/SMA, respectively.

Figure 13 shows the space charge distribution characteristics in the insulation layer of the coaxial cable model with four materials used as insulators. POE shows a negative effect on the space charge properties of the materials, while SMA can improve the space charge properties of the materials. The charge injection of the four groups of materials is mainly hole injection, which can be attributed to the temperature gradient in the insulation layer of the cable. Because the conductor core of the cable is the heat source of the system, the temperature rise is highest at the inner semi-conductive shield near the conductor core, and the Schottky current is proportional to the square of the temperature. Therefore, the charge injection at the inner semi-conductive shield is much higher than that at the outer semi-conductive shield.

**Figure 14:**
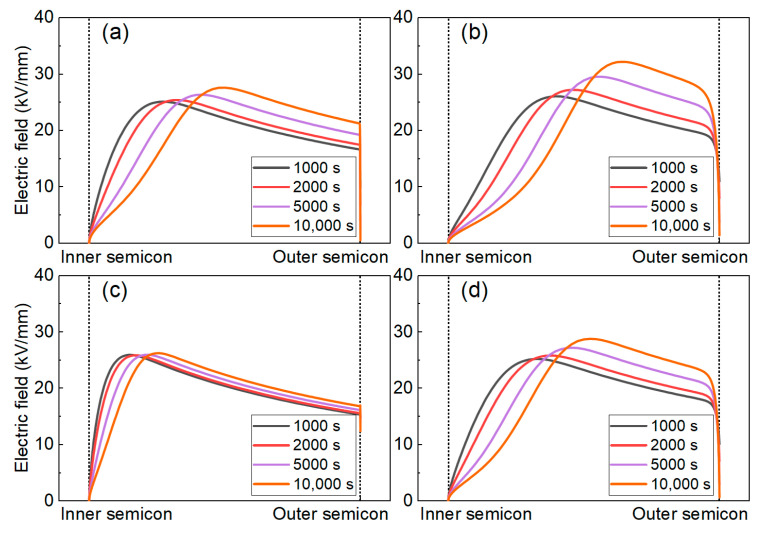
Simulation results of the electric field distribution in the cable insulation. (**a**–**d**) PP, PP/POE, PP/SMA, and PP/POE/SMA, respectively.

Figure 14 shows the electric field distribution of the cable insulation layer corresponding to the space charge distribution in Figure 13. It can be seen that the maximum field strength in each material moves from near the inner shield to the middle of the sample throughout the polarization process, which is consistent with the space charge moving behavior in Figure 12. The maximum electric field strength of the PP insulation is 27 kV/mm. After adding the POE, the electric field distortion in the PP/POE is more serious, reaching 32.5 kV/mm. After the introduction of SMA, the electric field distortion in the PP/SMA is significantly suppressed, and the maximum electric field is only 26 kV/mm. The maximum electric field of the PP/POE/SMA is less than 30 kV/mm, which shows a good effect of resisting electric field distortion.

## 4. Conclusions

This paper reports a potential organic deep trap additive, SMA, which can improve the DC electrical properties of both PP and PP/POE. The meso-morphology and crystalline morphology test results show that the introduction of SMA has little effect on the mesoscopic and macroscopic aggregate structures of PP-based materials. Moreover, the FT-IR results verify that SMA can be blended successfully into PP and PP/POE. The DC electrical test results indicate that SMA can improve the DC electrical properties of the composites, whether in the PP system or the PP/POE composite. After the introduction of SMA, the space charge and conductivity of the materials can be suppressed significantly. The breakdown strength of the PP/POE blended with SMA was enhanced from 158.8 kV/mm to 220.9 kV/mm at 70 °C, which exhibits an obvious improvement even at a high temperature. The TSDC results show that SMA can introduce deep traps with approximately 1.12 eV depth into the material. The results of the quantum chemical calculations are in agreement with those of the TSDC measurements. Based on the trap parameters obtained in TSDC, the BCT model was constructed for coaxial cable insulation, and the simulation results were consistent with the PEA test results. Furthermore, the distribution characteristics of the space charge in the cable insulation layer were simulated. The results show that SMA can significantly inhibit the space charge and resist the electric field distortion. In summary, PP composites modified by SMA can be a promising material for next-generation recyclable HVDC insulation.

## Figures and Tables

**Table 1:** The compounds of PP and modified PP.

	PP (wt %)	POE (wt %)	SMA (wt %)
PP	100	--	--
PP/POE	70	30	--
PP/SMA	99.5	--	0.5
PP/POE/SMA	69.5	30	0.5

**Table 2:** The parameters of the carrier transport simulation.

Type	Parameter	Value
Schottky emission	Electron injection barrier	1.27 eV
	Hole injection barrier	1.30 eV
Environment	Permittivity	2.3
	Inner temperature	70 °C
	Outer temperature	50 °C
	Thickness	31 mm

**Table 3:** Characteristic parameters of breakdown strength.

	PP		PP/POE		PP/SMA		PP/POE/SMA	
	*E*_b_/(kV/mm)	*β*	*E*_b_/(kV/mm)	*β*	*E*_b_/(kV/mm)	*β*	*E*_b_/(kV/mm)	*β*
30 °C	339.2	16.9	291.5	12.6	356.3	21.6	301.5	15.7
50 °C	295.6	15.8	248.8	18.8	348.7	19.0	281.1	18.5
70 °C	258.8	11.9	158.8	9.1	305.2	14.2	220.9	8.6

**Table 4:** Trap parameters of PP and modified PP.

Parameter	PP	PP/POE	PP/SMA	PP/POE/SMA
Electron mobility barrier (eV)	0.9	0.75	0.9	0.75
Hole mobility barrier (eV)	0.9	0.75	0.9	0.75
Electron trap depth (eV)	1.09	1.09	1.12	1.12
Hole trap depth (eV)	1.09	1.09	1.12	1.12

## Data Availability

The raw/processed data required to reproduce these findings are available from the corresponding author upon request.
